# Clinical application of CT-guided ^125^I seed interstitial implantation for local recurrent rectal carcinoma

**DOI:** 10.1186/1748-717X-6-138

**Published:** 2011-10-18

**Authors:** Zhongmin Wang, Jian Lu, Lin Liu, Tao Liu, Kemin Chen, Fenju Liu, Gang Huang

**Affiliations:** 1 Department of Nuclear Medicine, Renji hospital, Shanghai Jiaotong University School of Medicine, 1630 Dongfang Road, Shanghai, 200127, China; 2 Department of Radiology, Ruijin Hospital Luwan Branch, Shanghai Jiaotong University School of Medicine, Shanghai, 200020, China; 3 Department of General Surgery, Shanghai Ruijin Hospital Luwan Branch, Shanghai Jiaotong University School of Medicine, Shanghai, 200020, China; 4 Department of Radiology, Ruijin Hospital, Shanghai Jiaotong University School of Medicine, Shanghai, 200025, China; 5 School of Radiation Medicine and Public Health, Soochow University, Suzhou, 215123, China

## Abstract

**Purpose:**

The present study aimed to explore the safety profile and clinical efficacy of CT-guided radioactive seed implantation in treating local recurrent rectal carcinoma.

**Materials and methods:**

CT-guided ^125^I seed implantation was carried out in 20 patients with locally recurrent rectal carcinoma. 14 of the 20 patient had prior adjuvant external-beam radiation therapy (EBRT). The treatment planning system (TPS) was used preoperatively to reconstruct three dimensional images of the tumor and to calculate the estimated seed number and distribution. The median matched peripheral dose (MPD) was 120 Gy (range, 100-160 Gy).

**Results:**

Of the 20 patients, 12 were male, 8 were female, and ages ranged from 38 to 78, with a median age of 62. Duration of follow-up was 3-34 months. The response rate of pain relief was 85% (17/20). Repeat CT scan 2 months following the procedure revealed complete response (CR) of the tumor in 2 patients, partial response (PR) in 13 patients, stable disease (SD) in 3 patients, and progressive disease (PD) in 2 patients. 75% of patients had either CR or PR. Median survival time was 18.8 months (95% CI: 3.5-22.4 months). 1 and 2 year survival rates were 75% and 25%, respectively. 4 patients died of recurrent tumor; 4 patients died of distant metastases; 9 patients died of recurrent tumor and distant metastases. 3 patients survived after 2 year follow up. Two patients were found to have mild hematochezia, which was reversible with symptomatic management.

**Conclusion:**

CT-guided ^125^I seed implantation appeared to be a safe, useful and less complicated interventional treatment option for local recurrent rectal carcinoma.

## Introduction

Post-operative chemotherapy and external-beam radiation therapy (EBRT) is the standard adjuvant treatment for high-risk resected rectal carcinoma [1]. Despite the effectiveness of combined adjuvant therapies, local recurrence remains a significant problem in the 10-15% of high-risk patients who subsequently experience local relapse [2,3]. Successful surgical salvage of pelvic relapses is restricted to anastomotic recurrences and some centrally located relapses. In fact, more than 3 of 4 locally recurrent tumors cannot be resected completely [4,5]. Palliative surgical resection without additional therapy has resulted in a 3-yr survival of 8% with no 5-yr survivors in a recent Mayo Clinic series [6]. Interstitial implantation of ^125^I seeds into the tumor delivers a high dose of radiation to the tumor (range 140-180 Gy) by a very sharp fall-off containing the implanted volume, thus sparing nearby normal tissues. In addition, ^125^I seed has a slow continuous release of radiation (initial dose rate 0.07-0.09 Gy/h) that is radiobiologically advantageous, allowing repair of sublethal damage and reoxygenation of hypoxic areas in the late-responding tissues [7]. Therefore, radioactive ^125^I seed implantation is another choice for treatment of malignant tumors, which is widely applied for its curative effect, minimal surgical trauma, and few complications [8-15]. At our institution, we utilize ^125^I seed replacement routinely in recurrent tumors in various sites, which led us to investigate whether CT-guided brachytherapy is also feasible for rectal lesions. Therefore, we report here the results for 20 locally recurrent rectal cancer patients following ^125^I seed implantation.

## Materials and methods

### Patients

This retrospective study was approved by the hospital ethics committee, and all patients provided written informed consent. Between October 2006 and August 2009, 20 consecutive patients with locally recurrent rectal carcinoma (12 men and 8 women, mean age 63.5 years) were included in this retrospective, nonrandomized study. Of these patients, 3 had a tumor in the rectal upper third, 4 had a tumor in the rectal middle third, and 13 had a tumor in the rectal lower third. Median diameter of the tumor was 4.1 cm. All patients had undergone prior surgeries. Of the 20 patients, 10 had had both prior adjuvant external-beam radiation therapy (EBRT) and chemotherapy, 4 had received EBRT alone, and 2 had undergone adjuvant chemotherapy alone. The other 4 patients could not tolerate or refuse to have EBRT or adjuvant chemotherapy. The median EBRT dose applied in the adjuvant setting was 60 Gy (range 50-70 Gy). Patient characteristics are listed in Table 1. Patients were first diagnosed by using computed tomography (CT) or magnetic resonance imaging (MRI). Histological confirmation of the diagnosis was achieved in all 20 cases by CT-guided fine needle aspiration (FNA) 1 week before implantation. FNA has been accepted as a gold standard in the diagnosis of tumor cytology [16-20]. Furthermore, 9 patients had elevated serum carcinoembryonic antigen (CEA) level.

**Table 1:** Patient characteristics

Characteristic	n	%
Number of patients	20	100
Median age (range)	63.5(41-87)	
Gender		
Male	12	60
Female	8	40
Pathology		
Adenocarcinoma	18	90
Undifferentiated carcinoma	1	5
Malignant hemangiopericytoma	1	5
Tumor stage		
T2N0M	6	30
T2N1M0	2	10
T3N1M0	9	45
T3N2M0	2	10
T4N0M0	1	5
Tumor site		
Upper third	3	15
Middle third	4	20
Lower third	13	65
Location		
Presacral region	7	35
Anastomotic junction without expansion to the sacrum	13	65
Prior treatments		
Surgery	20	100
Miles	13	65
Dixon	7	35
TME	0	0
Radiation therapy & chemotherapy	10	50
Chemotherapy alone	2	10
Radiation therapy alone	4	20
none	4	20

### Instruments

**Figure 1:**
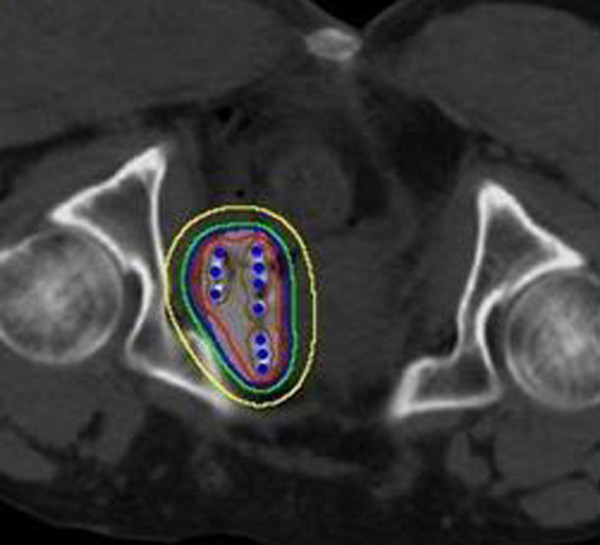
**A typical transverse slice showing the distributions of **^**125**^**I seeds and isodose curves after seed implantation from CT scan**. The isodose lines shown are 300 Gy (brown), 160 Gy (red), 100 Gy (blue), 80 Gy (green) and 40 Gy (yellow). PTV and ^125^I seeds are shown in light grey circle and blue dots, respectively.

We used a Siemens CT scanner with pelvic imaging conditions of 120 kV, 275 mA, and width of 5 mm. Dose distribution was calculated using a Fudan TPS2.00 brachytherapy planning system (Fudan University, Shanghai, China) based on the American Association of Physicists in Medicine TG43 brachytherapy formalism [21]. A series CT images are entered to reconstruct the three-dimensional graph of tumor; tumor volume is calculated and the three-dimensional graphs are displayed by the system. Figure 1 showed the distributions of ^125^I seeds and isodose curves after seed implantation from CT scan, which was analyzed by Fudan TPS 2.00. The ^125^I sealed seed sources were supplied by XinKe Pharmaceutical Ltd, Shanghai. For the ^125^I seed implantation we used 18G implantation needles and a turntable implantation gun (XinKe Pharmaceutical Ltd, Shanghai, China). The ^125^I seeds were manufactured from silver rods, which absorb ^125^I, and were enclosed in a titanium capsule welded by laser (XinKe Pharmaceutical Ltd, Shanghai, China). The diameter of each seed was 0.8 mm, the length was 4.5 mm, and thickness of the wall of the titanium capsule was 0.05 mm. ^125^I produces gamma rays (5% of 35 keV, 95% of 28 keV) with a half-life of 59.6 days, half-value thickness of 0.025 mm of lead, penetration of 17 mm, incipient rate of 7 cGy/h, and activities of 0.6-0.8 mCi.

### CT-guided implantation protocol

One week prior to seeds implantation, all patients underwent a detailed tumor volume study using CT scans with the thickness of 5 mm. The radiation oncologist outlined the gross tumor volume (GTV) and areas at risk for subclinical disease on each transverse image. The planning target volume (PTV) included the entire GTV and 0.5-1.0 cm margins. The dose was prescribed as the minimal peripheral dose (MPD) encompassing the PTV. The median MPD was 120 Gy (range 100-160 Gy). The distribution and MPD of ^125^I seeds were calculated using computerized treatment planning system. To reach the maximum radiation effect, the number of seeds implanted was 15% more than needed. Implantation was guided by CT according to our TPS. ^125^I with a nominal activity of 0.6-0.8 mCi/seed and a diameter of less than 1 mm was used as a radiation source and implanted into the recurrent rectal tumor under fluoroscopy CT guidance, at a spacing of 1 cm, avoiding puncturing vessels, urinary bladder, and other nearby structures. Implant parameters are listed in Table 2. Patients were placed on a clear liquid diet 24 hours prior to the implantation. All the brachytherapy implants were performed in a standard CT room under local anesthesia. CT imaging was taken at intervals of 5 mm. The distance between the adjacent implantation needles was approximately 1 cm. Repeat CT with the implantation needles in place permitted adjustment of depth and angle of needle direction. Two to five seeds per needle were loaded, and seeds were released 0.5-1 cm apart upon withdrawing the needles. Patients were kept in our radiooncology/interventional ward for 2 full days.

**Table 2:** Implant parameters

Characteristic	Median	Range
Volume implanted (cm3)	68.9	26.9-97.3
MPD (Gy)	120	100-160
Dose rate (Gy/h)	0.07	0.05-0.09
Total activity (mCi)	18.2	9.5-66.6
No. seeds	58	19-111
Activity/seed (mCi)	0.7	0.6-0.8

### Clinical benefit response (CBR)

Clinical benefit response was derived from measurement of pain, functional impairment (assessed by KPS), and weight loss [22]. For patients to achieve an overall rating of positive CBR, they had to be positive for at least one parameter (pain, performance, status, or weight) without being negative for any of the others [23]. This improvement had to last for at least 4 weeks. Visual analog scale (VAS) pain score was recorded as level 0 to 10, in which 0 indicated no pain, 1 to 3 indicated mild pain, 4 to 7 meant moderate pain, and 8 to 10 severe pain. Scoring began after the ^125^I seeds were implanted.

### Evaluation of curative effect

Patients were examined by CT 2 months after the operation. The efficacy was determined according to the tumor response standards suggested by the World Health Organization [24]. Briefly, complete response (CR) was defined as the complete disappearance of the lesion lasting for more than 4 weeks. Partial response (PR) referred to the situation where the size (i.e., the longest dimension multiplied by maximal upright dimension) of the lesion decreased by more than 50% and then remained unchanged for 4 weeks. Stable disease (SD) was defined as the situation where the size of the tumor decreased by less than 50% or increased by less than 25%. Response rate was defined as the sum of CR and PR. Local tumor control after brachytherapy was defined as the absence of tumor progression in CT (SD+PR+CR). Serum CEA level was checked every month post-implantation as an indicator of prognosis.

### Postimplant chemotherapy and radiation therapy

Three out of 20 patients who gave consent to chemotherapy received combined treatment with Oxaliplatin 100 mg/m^2 ^and 5-fluorouracil (5-FU) 125 mg/m^2 ^1 week after implantation. The chemotherapy was a 2-day schedule which contained Oxaliplatin on the first day followed by 2 days of 5-FU. The chemotherapy was repeated every 3 weeks for up to six cycles if tolerated. Post-implantation EBRT was generally recommended for previously unirradiated patients; however, no patient received EBRT if they had post-operative complications or were unable to receive the planned EBRT or refused further therapy.

### Follow-up

All 20 patients entered the follow-up phase immediately after the implantation. The intended follow-up period was 34 months, with first visits at 1 month post intervention, and every 3 months subsequently for clinical examination, blood sampling, and imaging studies, including chest X-ray, abdominal and pelvic CT scans and ultrasonography, performed earlier if a new clinical sign or symptom appeared. No patients were lost to follow-up. Follow-up pelvic CT scans to evaluate response were obtained on all patients at various time intervals from implantation.

### Statistical analysis

With SPSS 13.0 software, quantitative indicators before and after the operation were compared using paired *t *test or nonparametric methods. The median survival time of survival analysis was evaluated by the Wilcoxon test and Kaplan-Meier methods. A *P *value of less than 0.05 was defined as statistically significant.

## Results

### Effects on cancer-associated Karnofsky and pain physical score

Karnofsky score increased dramatically post-^125^I implantation as indicated in nutritional status, sleeping time, and functional level (P < 0.05). Thus the overall quality of life improved. Details are listed in Table 3.

**Table 3:** Change of Karnofsky physical score

	100	90	80	70	60
Pre-op	0 (0/20)	5.0 (1/20)	5.0 (1/20)	40.0(8/20)	50.0 (10/20)
Pre-op	0 (0/20)	55.0 (11/20)	25.0 (5/20)	15.0 (3/20)	5.0 (1/20)

Data are presented as % (cases)

### Pain relief

Symptoms of refractory pain were significantly resolved post-treatment (P < 0.05). In 20 patients, 11 (55.0%) patients had severe pain; 5 (25.0%) patients had moderate pain; 4 (20.0%) patients had mild pain before seeds implant. The response rate of pain relief was 85%. Change of pain score is shown in Table 4.

**Table 4:** Change of pain score

	No pain (%)	Mild pain (%)	Moderate pain (%)	Severe pain (%)
Pre-op	0 (0/20)	20.0 (4/20	25.0 (5/20)	55.0 (11/20)
Post-op	30.0 (6/20)	40.0 (8/20)	15.0 (3/20)	15.0 (3/20)

Data are presented as % (cases)

### Response to treatment

**Figure 2:**
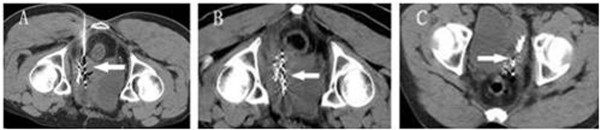
**Locally recurrent rectal cancer with **^**125**^**I seeds implant under CT guidance**. (a) CT scan shows that ^125^I seeds are implanted into the tumor via 18G implantation needles (arrow). (b) CT scan shows the distribution of ^125^I seeds post implantation (arrow). (c) 2 month follow-up. CT scan shows recurrent tumor partially decreased and ^125^I seeds gathered together (arrow).

**Figure 3:**
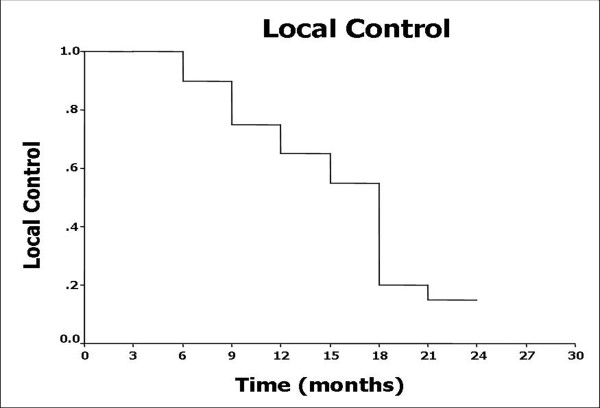
**Local control curve after CT-guided **^**125**^**I implantation**.

Tumor response, which was demonstrated on repeated CT film 3 months post-treatment, revealed complete response (CR) in 2 cases, partial response (PR) in 13 cases (Figure 2), stable disease (SD) in 3 cases, and progressive disease (PD) in 2 cases. Overall responding rate (CR+PR) for this group of patients was 75%. Local tumor control rate was 90%. 1- and 2-year local control rates were 65.0% and 15.0%, respectively (Figure 3). 4 cases died as a result of recurrent tumor progression; 4 cases died because of distant metastases; 9 cases died of recurrent tumor progression and distant metastases. 3 cases survived to end of the follow-up. Elevated serum CEA level before ^125^I seeds implantation were detected in 9 cases, which reduced in 5 of 9, no change in 3 of 9, and increased in 1 of 9 after 1-3 months.

### Overall survival

**Figure 4:**
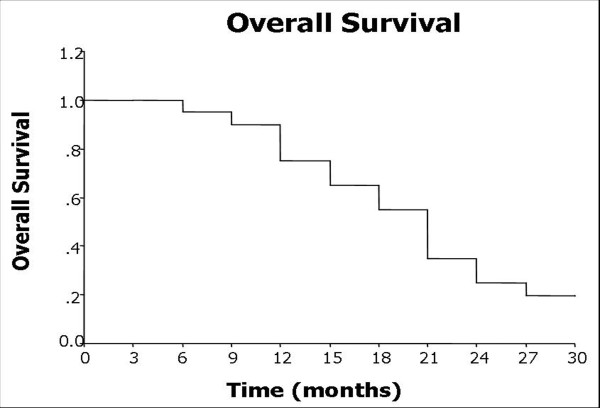
**Overall survival curve after CT-guided **^**125**^**I implantation**.

Follow-up period was 3 to 34 months, the median time was 22 months. 14 patients and 6 patients reached the one-year and two-year follow-up, respectively. Median survival time for all patients was 18.8 months (95% CI: 3.5-22.4 months). The estimated 1- and 2-year survival rates were 75% and 25%, respectively (Figure 4).

### Adverse reactions

There were no major complications such as fever, radiation enteritis, colorectal tract perforation and detected during our follow-up period. Two (10%) of twenty had grade one lower gastrointestinal reaction. The patients were found to have mild hematochezia, which was reversible with symptomatic management. All patients underwent an immediate post-procedure CT scan, no ^125^I seeds migrated to other tissues or organs. Bowel and rectal complaints were documented using a modified Radiation Therapy Oncology Group (RTOG) rectal symptom scoring scale [25].

## Discussion

Various therapeutic modalities have been applied in the treatment of local recurrent rectal cancer, such as surgery, radiotherapy and chemotherapy - either alone or in combination. A five-year disease-free survival rate of less than 10 percent has been reported after radiation therapy with or without chemotherapy, but radiation therapy alone or combined with chemotherapy can provide temporary relief of symptoms [26,27]. In patients who undergo radical surgery for rectal cancer, 4-33% develop locoregional relapse. Without treatment, these patients with local recurrent rectal cancer have a median survival of 8 months [28-31]. In non-fixed recurrent tumors, complete resection can be achieved with limited surgery such as abdomino-perineal resection and the outcomes are relatively favorable. 50 percent of patients with recurrent cancer after curative surgery have an isolated pelvic tumor which could be treated by salvage surgery. However, the actual salvage surgery rate ranges between 30 and 40 percent [32-34]. Our goals of treatment for locally recurrent rectal cancer are palliation of symptoms, better quality of life, and fewer treatment-related complications. CT-guided implantation of radioactive seeds might be an alternative in the treatment of local recurrent rectal carcinoma.

Percutaneous image-guided seed implantation, which can be performed without surgery or general anesthesia, has attracted increasing attention because of its ability to increase radiation dose to the region of interest without damaging neighboring organs [35]. Many published work [9-15] had reported percutaneous Iodine-125 seeds implantation as a sole modality showed promising results in the treatment of malignant tumor, such as recurrent rectal carcinoma, non-small cell lung cancer, recurrent soft tissue sarcomas, spinal metastatic and primary paraspinal malignancies, recurrent head and neck cancers, and so on. With this technique, highly effective radiation doses are applied as a single fraction, ensuring protracted cell killing over a period of up to several weeks or months.

Lefevre et al. [36] reported a series of 8 patients with local recurrence of pelvic cancer treated with radiofrequency ablation (RFA), complications occurred in six patients including minor pain, ureteric obstruction and colo-vesical fistula. It allowed good symptom control in patients with pain but morbidity was high. Ohhigashi et al. [37,38] published a small series of 10 patients to evaluate RF ablation as new local treatment for recurrence of rectal carcinoma. Tumor smaller than 3 cm were treated with a single electrode and those greater than 3 cm in diameter required multiple insertions. The authors proposed that RF ablation is an effective treatment for tumor less than 4 cm in diameter that are not in close proximity to major blood vessels. Shimizu et al. [39] reported nine recurrent pelvic lesions in 8 patients after curative resection of rectal cancer were treated with real-time magnetic resonance-guided microwave coagulation therapy (MCT). Local re-recurrence in the ablated lesion was observed in 2 of 9 lesions. The 3-year overall survival rate was 12.5% (1/8 patients). Compared with other interventional procedures, advantages include interference-free and accurately predictable energy distribution, treatable size of a target lesion, and lower rate of acute adverse effects due to maintenance of tissue continuity. Extensive experience with this technique had been collected in several preceding studies targeting pancreatic malignancies as well as one study targeting lung malignancies [8,40,41]. However, there are few reports on CT guided implantation of radioactive seeds in the treatment of recurrent rectal carcinoma [42]. At our institution, the most commonly used isotope is ^125^I, and ^125^I placement has become a routine treatment for recurrent tumors at various sites. Therefore, we here present the results of 20 local recurrent rectal carcinoma patients following ^125^I seeds implantation.

Possible advantages of ^125^I seeds over other forms of radiotherapy are as follows: 1. Radiation from seeds is characterized by attenuation over a short distance, which can keep a higher accumulative dose (up to 160 Gy) within the tumor. 2. ^125^I seeds can kill tumor cells continually by keeping cells in the resting period and causing tumor stem cell apoptosis. 3. Deficiency of oxygen is a bottleneck of conventional external radiotherapy.

Does et al. first reported 30 local recurrent rectal cancer patients treated with brachytherapy [43]. Mean follow-up and local control for gross residual disease and microscopic residual disease were 26.5 months and 37.5 percent versus 34 months and 66 percent. Eighteen patients (64%) had local recurrent rectal cancer under control in his series. Martinez et al. reported a similar experience in 29 patients with colorectal adenocarcinoma recurrent in the pelvis treated with permanent ^125^I seed implantation [44]. All patients had undergone prior surgery, and 72% of them had prior EBRT. The implanted residual tumor volume was microscopic in 38% and gross in 62%. The implanted area received a median minimal peripheral dose of 140 Gy to total decay. The 1-year, 2-year, and 4-year actuarial locoregional control rates were 38%, 17%, and 17%, respectively, with a median time to local failure of 11 months. The median overall survival rate was 18 month.

Compared to intraoperative brachytherapy, the percutaneous implantation of ^125^I seeds used in our series may have certain advantages, such as better placement of seeds using image guidance and more precise seed distribution in target volume. In our group of patients we implanted ^125^I seeds under CT guidance and repeated CT after implantation which showed even distribution of radioactive seeds. Overall response rate of 75%, local control rate of 90%, and pain relief rate of 85% demonstrated promising clinical outcomes in this group. Follow-up period was 2 to 34 months. Median survival time for all patients was 18.8 months (95% CI: 3.5-22.4 months). The estimated 1- and 2-year survival rates were 75% and 25%, respectively. These data suggest that ^125^I seed implantation under CT guidance has the potential to improve local control of recurrent rectal cancer.

Koutrouvelis reported a case of CT-guided salvage brachytherapy of recurrent colorectal cancer in the pelvis [45] in a patient whose initial CEA was 67.0 ng/mL. After successful Iodine-125 seed implantation the CEA level dropped to 5.7 ng/mL 12 months later. In our series, nine patients had elevated serum CEA level before implantation. 1-3 months following implantation, the CEA was reduced in 5 patients, unchanged in three and increased in one. Thus, monitoring of sequential serum CEA levels pre- and post-implantation with local recurrent rectal cancer might help evaluate treatment responses.

Finally, there were fewer complications compared with other interventional ablation procedures. From these data, it appeared that ^125^I seed implantation of recurrent rectal tumors offered both control of the primary tumor and significant palliation of symptoms.

In conclusion, this study suggests that CT-guided brachytherapy using ^125^I seeds implantation is safe, useful and uncomplicated and could produce significant pain relief for treating local recurrent rectal carcinoma. The long-term effectiveness of this treatment modality is still under investigation.

## Competing interests

The authors declare that they have no competing interests.

## Authors' contributions

ZMW carried out data acquisition, performed the statistical analysis, drafted the manuscript and participated in the sequence alignment. JL participated in the design of the study and participated in the sequence alignment. LL, TL and KMC participated in the sequence alignment. FJL carried out data acquisition. GH conceived of the study, and participated in its design and coordination and helped to draft the manuscript. All authors read and approved the final manuscript.
